# Exploring Near- and Far-Field Effects in Photoplethysmography Signals Across Different Source–Detector Distances

**DOI:** 10.3390/s25010099

**Published:** 2024-12-27

**Authors:** Ángel Solé Morillo, Joan Lambert Cause, Kevin De Pauw, Bruno da Silva, Johan Stiens

**Affiliations:** 1 Department of Electronics and Informatics (ETRO), Vrije Universiteit Brussel (VUB), 1050 Brussels, Belgium; jlambert@etrovub.be (J.L.C.); bruno.da.silva@vub.be (B.d.S.); jstiens@etrovub.be (J.S.); 2 Department of Biomedical Engineering, Universidad de Oriente, Santiago de Cuba 90500, Cuba; 3 Human Physiology and Sports Physiotherapy Research Group, Vrije Universiteit Brussel, 1050 Brussels, Belgium; kevin.de.pauw@vub.be; 4 Brussels Human Robotics Research Center (BruBotics), Vrije Universiteit Brussel, 1050 Brussels, Belgium

**Keywords:** photoplethysmography, PPG, influencing factors, instrumentation, source–detector distance, near-field, far-field, signal quality index

## Abstract

Photoplethysmography is a widely used optical technique to extract physiological information non-invasively. Despite its large use and adoption, multiple factors influence the signal shape and quality, including the instrumentation used. This work analyzes the variability of the DC component of the PPG signal at three source–detector distances (6 mm, 9 mm, and 12 mm) using green, red, and infrared light and four photodiodes per distance. The coefficient of variation (CV) is proposed as a new signal quality index (SQI) to evaluate signal variabilities. This study first characterizes the PPG system, which is then used to acquire PPG signals in the chest of 14 healthy participants. Results show a great DC variability at 6 mm, homogenizing at 9 and 12 mm. This suggests that PPG systems are also sensitive to the near- and far-field effects commonly reported and studied in optics, which can impact the accuracy of physiological parameters dependent on the DC component, such as oxygen saturation (SpO_2_).

## 1. Introduction

Photoplethysmography (PPG) is a non-invasive optical technique used to measure blood volume changes in the skin’s microvasculature caused by the pulsatile nature of the circulatory system [1]. PPG is widely used, as it is the fundamental principle behind pulse oximeters, the standard of care since the late 1980s for peripheral oxygen saturation (SpO_2_) during anesthesia [2]. Since then, the number of PPG uses and applications has only increased, especially in recent years, due to the rapid expansion of health wearables, which primarily integrate PPG to estimate heart rate, among other physiological parameters [3].

The generated PPG signal comprises a pulsatile waveform (called ‘AC-PPG’) that reflects these blood volume changes synchronized with each heartbeat. The AC-PPG is superimposed on a slowly varying baseline waveform (‘DC-PPG’). This DC component corresponds to the light absorption by non-pulsatile components, such as bone, muscle, melanin, or venous blood, and it contains valuable information about respiration, venous flow, sympathetic nervous system activities, and thermoregulation [4].

The PPG waveform is used to extract signal features and estimate physiological parameters [5,6]. Nevertheless, a wide range of influencing factors can induce a change in this signal shape. These factors can be divided into three main categories: subject-dependent, physiological, and acquisition. The measurement conditions will determine the relevance of each of them, which will add up together to generate the ‘final’ signal shape. While some of these factors will be used to detect a targeted physiological change (e.g., a blood pressure change), some others will act as a source of inaccuracy, which can lead to the misestimation of the physiological information [7,8,9].

As part of the acquisition factors, the electronic instrumentation selected to acquire PPG signals also influences the PPG waveform [8,10]. The essential instrumentation elements needed to acquire PPG signals include an LED as the light source, a PIN-photodiode for detecting light, and a trans-impedance amplifier (TIA) to convert the photodiode’s current signal into a measurable voltage [11]. Pulse oximeters use red and infrared LEDs and a single photodiode to measure heart rate and blood oxygen levels. More advanced systems, known as multi-wavelength PPG, integrate additional wavelengths and may include multiple sources and detectors [11]. The optical front-end configuration of these systems, i.e., the number of LEDs, photodetectors, their relative distance, and spatial distribution, can significantly impact the PPG signal [8]. It is important to characterize and calibrate the chosen components and their distribution before use. If the instrumentation influence is not properly accounted for, signal changes caused by it (e.g., DC offset) could be wrongly associated with other (physiological) sources, reducing the accuracy and performance of PPG applications.

The LED properties must be included as part of the instrumentation characterization. The impact of the LED wavelength has been broadly evaluated in many studies covering multi-wavelength PPG applications [12,13,14]. The LED viewing angle, i.e., the luminous aperture, also influences the PPG signal, as recently reported by our group [15].

Another LED characteristic is the intrinsic LED light generation and emission, which is not ideal and gives rise to near- and far-field regions, well known and studied in optics science [16]. Although PPG is an optical technique, there appears to be no existing published research on the effects of near- and far-field influences on PPG signal acquisition.

The main goal of this work is to determine if near-field inhomogeneities affect PPG signal acquisition and to identify where the stable far-field begins for PPG applications. Additionally, this study examines the impact of these near- and far-field effects when using coated versus uncoated optical windows. These windows are the protective material to isolate the electronics from the user while guaranteeing optimal light transmission. As reported already in [15], due to Fresnel reflections, uncoated glasses are subject to undesired light reflections, which can be minimized if anti-reflection coatings are used.

The near- and far-field influence on a custom PPG system is investigated by exploring three source–detector distances (6 mm, 9 mm, and 12 mm) and utilizing four evenly spaced photodiodes per distance to create different source–detector pairs for each distance using green, red, and infrared light. The coefficient of variation (CV) is used as a signal quality index (SQI) to evaluate the influence of the near- and far-fields, as it assesses the degree of agreement among the photodiodes readout per source–detector distance.

The system is first evaluated in a lab setting using an optical setup, followed by PPG signal acquisition in healthy participants in the chest region. The chest region is used due to its large measurement area, which is very suitable for obtaining PPG signals across the different source–detector pairs. This study focuses on changes in the DC-PPG, the signal component that could be affected by these effects, and not on the AC-PPG, which is caused by blood volume changes in the skin. To analyze the possible impact of near-field effects on the physiological parameters, the AC-PPG and DC-PPG signals recorded with each photodiode using red and infrared light are employed to estimate SpO_2_ values.

The main contributions of this work are:

1. Identification of near-field effects in PPG systems in reflection mode.
2. Methodology to identify the start of the stable far-field region of LEDs.

This paper is organized as follows: Section 2 covers the related work regarding LED light emission; Section 3 describes the materials and methods used for the study; Section 4 presents the results; and Section 5 discusses them. This work ends with general conclusions in Section 6.

## 2. Background and Related Work

### LED Light Emission Characteristics

**Figure 1:**
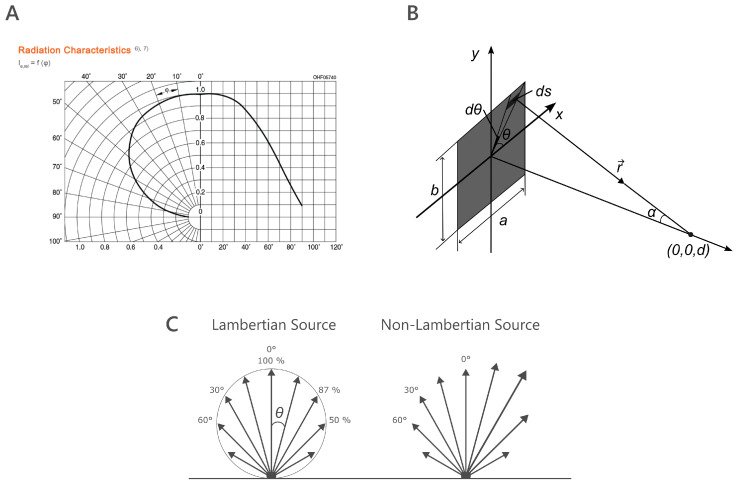
(**A**) Radiation curve of SFH 7013 datasheet [20]. (**B**) Diagram of radiance in the far field using a rectangular light source. Adapted with permission from Optica Publishing Group [17]. (**C**) Radiance pattern of a Lambertian source versus a non-Lambertian source.

When LEDs are browsed and selected for PPG systems, the developer normally looks at the LED datasheet to find all the relevant technical specifications. These specifications include a figure in polar and Cartesian coordinates with the radiation characteristics of the LED, which indicates the relative radiant intensity variation as a function of the emitting angle (Figure 1A). Light intensity is maximal at 0° from the source vertical axis, and it decreases linearly with increasing angle θ.

Theoretically, this intensity definition only applies to a point source, mathematically defined as the intersection between two lines with no size (i.e., no width, no length, and no depth). LEDs do not meet this definition, as the active part has an area (typically under 1 mm^2^). In practice, LEDs are considered point sources when their generated radiation is detected from a distance much larger than the source dimensions. This working distance is known as ‘far-field’ and is typically achieved when the distance (*d*) is five times larger than the source dimensions (Figure 1B) [16]. In the far field, LEDs are considered a Lambertian source (Figure 1C), where radiance is uniform in all directions and its intensity drops following the cosine of θ. Here, the radiation pattern stabilizes, allowing for simpler geometric models. Light intensity depends primarily on the emission angle for a given distance, as shown in datasheets, and it decreases inversely as the square of the distance [16,17].

When this condition is not met, the working distance is known as ‘near-field’. In the near-field, however, the LED cannot be approximated as a point source, and the light’s spatial distribution no longer varies linearly with distance and angle. This region exhibits non-uniform intensity profiles, influenced by the size of the LED and its optical packaging, and where the typical radiation curves found in datasheets are not applicable [18]. In an LED, light is generated through spontaneous emission in the light-emitting layer, which results in incoherent light, meaning its intensity is the linear sum of emissions from all points on the LED surface. While the active region behaves almost like a Lambertian light source, the light undergoes transformations as it passes through various materials and surfaces within the LED. Each emission point on the surface may not strictly follow Lambertian behavior (Figure 1C). Additionally, special microstructures on the LED surface, such as microlens or internal waveguiding effects, can alter the emission direction and light cone [19].

The *5 times rule of thumb* (d=5·D) to determine the beginning of the far-field corresponds to a circular source, being *D* the source diameter. If the source is rectangular, as it is for LEDs, this distance is approximated to d≃5.74·D, with *D* being the side of the rectangle [16,17]. Given the light emission inhomogeneities caused by the LED package, this might need to be considered as the source size and not just the active area.

More complex models have also deepened into the analysis of near- and far-field effects, in particular, the irradiation pattern of LEDs in the near field [19], the near- and far-field behavior of LEDs arrays [16], or an in-depth analysis of far-field sub-regions [21].

PPG is an optical technique that uses LEDs as the light source to which these near- and far-field effects apply. In the near field, we could expect inhomogeneous light distribution, meaning that for a given source–detector distance, PPG signals measured from different points could have different DC amplitudes. In other words, near-field effects are a potential PPG influencing factor, which, to the author’s knowledge, has not been reported before.

## 3. Materials and Methods

To evaluate the influence of the near- and far-field effects on the PPG signal acquisition, first, a lab experiment was conducted using a diffuse reflector to homogeneously illuminate the optical front-end designed to have three different source–detector distances with four photodiodes for each distance. Afterward, the same optical front-end was used to acquire PPG signals and estimate SpO_2_ in the chest of 14 healthy participants.

### 3.1. Lab Experiment

**Figure 2:**
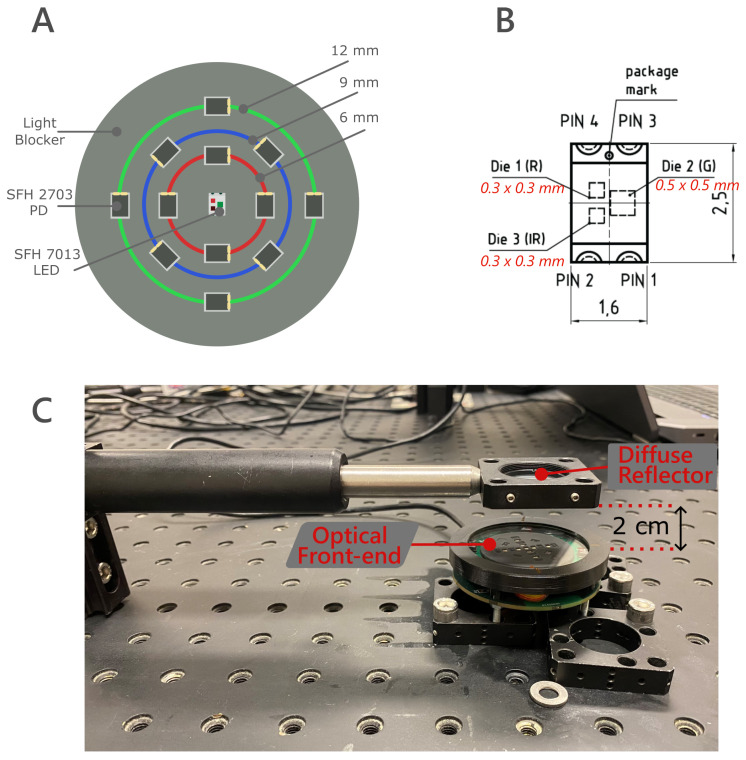
(**A**) Top view of optical front-end used for the near-field experiment. The multi-chip SFH 7013 LED is placed in the center with 12 SFH 2703 PIN photodiodes (PD) distributed in three rings of radius 6 mm, 9 mm, and 12 mm around it. A light blocker is placed to prevent direct light from the LEDs from reaching the photodiodes. (**B**) SFH 7013 dimensions, showing the active areas of the green (G), red (R), infrared (IR) LEDs, and the protective layer. From datasheet [20]. (**C**) Complete optical setup used for the near- and far-field experiment. A diffuse reflector is placed at 2 cm from the optical front-end to reflect light back into the photodiodes homogeneously.

Figure 2 illustrates the experimental setup used for the near- and far-field evaluation. A custom optical front-end was developed, featuring a central SFH 7013 multichip LED (OSRAM, Munich, Germany) [20]. The multichip LED includes green (550 nm), red (655 nm), and infrared (940 nm) LEDs, each with a half-angle of 65°. Surrounding the LED are twelve SFH 2703 PIN photodiodes (OSRAM), which have a spectral sensitivity range of 400–1100 nm. The photodiodes are arranged concentrically in three rings around the LED to achieve source–detector distances of 6 mm, 9 mm, and 12 mm. Each ring contains four evenly spaced photodiodes, resulting in four source–detector pairs per distance, with each photodiode detecting light from a different area within that source–detector distance (Figure 2A). For each source–detector distance, the output of the photodiodes will be compared to determine the near-field effects.

Figure 2B shows the exact dimensions of the multichip LED. The active areas of the red and infrared LEDs are 0.3 × 0.3 mm, while the green LED has an active area of 0.5 × 0.5 mm. The packaging size, excluding the soldering pads, is 1.6 × 1.6 mm. These dimensions are critical for understanding the near- and far-field effects, as both the active area and packaging size will be used to calculate the far-field’s theoretical start to better understand the packaging’s possible influence. A 3D-printed light blocker is mounted on the optical front-end to prevent direct light from reaching the photodiodes by guiding the LED light in the forward direction. With this, it is maximized that light interacts first with the skin or surface of interest, the diffuse reflector in this case, before reaching the photodiodes.

The LEDs are connected to a TLC5925 driver (Texas Instruments, Dallas, TX, USA), which controls the LED switching and driving current. The photodiodes are connected to a differential trans-impedance amplifier (TIA) circuit with a digital 1 MOhm variable potentiometer to control the amplification value. A 12 pF capacitor is placed in parallel for noise reduction and stability. The TIA configuration choices are based on a previously performed evaluation by our research group [10]. A Programmable System-on-Chip (PSoC) CY8CPROTO-063-BLE microcontroller is used to control the led driver, multiplex the photodiodes, and measure their readout using a 12-bit SAR ADC configured in differential mode at a sampling frequency of 125 Hz. A printed-circuit board (PCB) was designed to connect the optical front-end components with the PSoC, all housed in a 3D-printed casing. The case is designed to add optical windows to the front-end if needed. For this experiment, two 1 mm borosilicate protective glasses (UQG Optics, Cambridge, UK) were used, an uncoated one and a coated one in the visible–near infrared spectrum.

Figure 2C shows the complete optical setup assembled for the experiment. The optical front-end was fixed to an optical table, and a diffuse reflector (DG10-600-P01, Thorlabs, Newton, NJ, USA) was mounted 2 cm from it. A diffuse reflector reflects light uniformly following a Gaussian distribution. The selected diffuser is an uncoated N-BK7 polished glass with 600 grit, achieving an intermediate diffusion pattern over the 350–2000 nm range. The distance from the optical front-end guarantees that the diffusion pattern is generated before the light reaches the optical front-end. The diffuser was aligned with the center of the optical front-end, i.e., the LED, so all photodiodes in a given ring are illuminated equally, following the diffuser’s Gaussian distribution.

The green, red, and infrared LEDs were individually turned on, setting a 5 mA driving current. The diffuse reflector reflected back the generated light into the optical front-end, and the readout of all the photodiodes was measured for 10 s for each LED. An additional measurement was taken with the LEDs off for reference. This was performed three times in a dark room, one for each optical window configuration: no glass, uncoated glass, and coated glass.

The recorded DC values were averaged in Matlab and normalized, taking the highest DC value registered as 1. The coefficient of variation (CV) was employed to measure the dispersion or variability in the DC values recorded across quartets of photodiodes for each source–detector distance for all three wavelengths and optical windows. The CV for each quarter of photodiodes was calculated using the DC values (*data*) mean and standard deviation [22,23]:

(1) $$CV=\frac{std(data)}{mean(data)}*100$$

The CV is particularly useful in this context, as it normalizes the standard deviation relative to the mean, enabling comparisons across different source–detector distances, wavelengths, and optical windows, which can give different DC offset values. This way, the CV provides an indirect way of assessing near-field and far-field effects, as these phenomena influence the variability of measured DC values for a given quartet of photodiodes. In the near field, light propagation is affected by inhomogeneous light emission, potentially leading to higher CVs, while in far-field configurations, homogenized light emission may reduce the variability.

The CV was chosen over other variability metrics because it accounts for relative variability in a way that absolute measures do not. This study needs this normalization since the mean signal intensities (DC values) can vary significantly across photodiode quartets. Alternative metrics such as the standard deviation or range (max–min) could have been used but lack the normalization feature of CV. For instance, the standard deviation is an absolute measure of variability that does not scale with the mean, making it harder to interpret in datasets where mean values differ substantially.

In this context, the coefficient of variation can be understood as a signal quality index metric for near-field effects. SQIs are used in PPG applications to produce measures of PPG signal quality. Multiple PPG pulse wave characteristics have been used to assess signal quality, including amplitude, shape, and timing characteristics, higher-order statistics, template matching, visual annotation, or machine learning [5,24,25,26,27]. The SQI choice is based on the application and noise source. They aim to identify and eliminate noise-corrupted signals from the analysis, allowing a reliable estimation of physiological parameters [5]. Near-field effects can be understood as a noise source that can be measured by comparing the signal agreement among the set of four photodiodes per source–detector distance using the CV.

Once signal quality indices are extracted, the indices’ values must be related to high- and low-quality PPG signals. Decision rules are then established to determine the labeling of the PPG signal [26]. In this study, the CV calculated with the LEDs off will be taken as the reference variability, expected to be minimum (<5%), to assess the presence of near-field effects.

### 3.2. PPG Signal Acquisition

The same PPG system using the uncoated glass was employed to acquire PPG signals in 14 healthy participants. This study was conducted in compliance with the Declaration of Helsinki and was approved by the Ethics Committee for Human Sciences (ECHW) of the Vrije Universiteit Brussel (VUB). All participants provided informed consent, and age, gender, body mass index, skin tone following the monk skin tone (MST) scale, body temperature, pulse rate, and SpO_2_ were obtained as baseline demographic data, summarized in Table 1. A digital thermometer was used to measure body temperature in the armpit, and a finger pulse oximeter (MightySat Rx, Masimo, Irvine, CA, USA) was used for pulse rate and SpO_2_.

**Figure 3:**
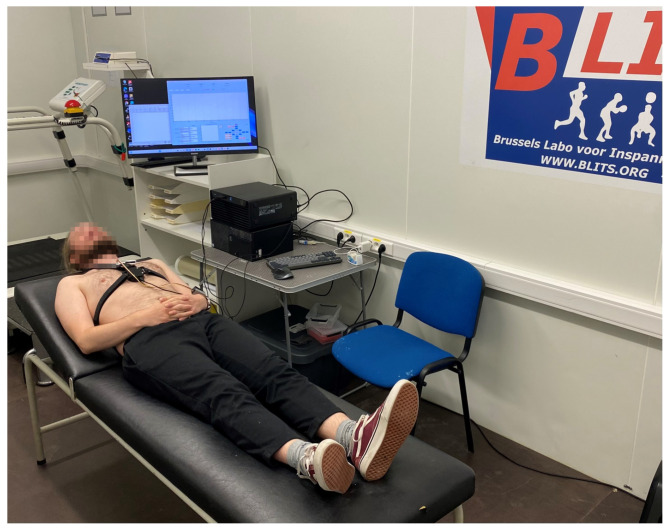
Setup of in-vivo investigation performed in a controlled climatic chamber. *Photo used with permission from the subject*.

Measurements were taken in a climate-controlled room at 22 °C and 45% relative humidity in the *Brussels Laboratory for Exercise and TopSport* (BLITS) of the MFYS research group at the VUB. Measurements were conducted with subjects lying on a bed in a supine position, instructed to relax and avoid body movements as much as possible (Figure 3). The PPG system was attached to the participant’s chest, as shown in the figure. Raw PPG signals were collected using green, red, and infrared light for three minutes, setting a 5 mA driving current. The photodiode readouts with the LEDs off were also measured to detect ambient light and eliminate its influence on the PPG signal. The PPG-system analog front-end gain was tuned for each subject, maximizing the PPG signal amplitude but avoiding saturation in any channel. To achieve this, the TIA feedback resistance was modified in the 150–250 KOhms range.

During post-processing in Matlab, the PPG signals were first low-pass filtered with a digital infinite impulse response (IIR) filter Chebyshev order four and a cutoff frequency of 2 Hz to eliminate possible high-frequency noise sources. A window of at least two minutes was manually selected to guarantee a stable PPG signal without undesired motion artifacts. The DC-PPG component was calculated by subtracting the average of the AC-PPG lower peaks from the measured ambient light value with the corresponding photodiode.

The DC-PPG’s coefficients of variation were calculated for each wavelength and source–detector distance combination. The CVs obtained with each subject are averaged and expressed as a mean±std.

To evaluate the impact of near-field effects in physiological parameters dependent on the DC-PPG component, the SpO_2_ was calculated with every photodiode. SpO_2_ is estimated using the R-ratio [28], which is defined as follows:

(2) $$R=\frac{{\left(AC/DC\right)}_{Red}}{{\left(AC/DC\right)}_{IR}}$$

R is the *ratio of ratios* obtained from the AC-PPG and DC-PPG components of the simultaneously measured red and infrared signals. The R-ratio is linked to SpO_2_ using a calibration curve determined empirically [28]. This method is used by clinical pulse oximeters, where each device’s calibration curve is established through a controlled desaturation study, following the ISO 80601-2-61:2017 standard [29]. Such a desaturation study could not be performed for this experiment, so the SpO_2_ values were estimated using the standard empirical equation $SpO_{2}\;=\;110\;-\;25R$ [28]. This formula is not valid for an accurate SpO_2_ estimation, but it is useful to assess how SpO_2_ values can be affected by near-field effects. The CVs for the SpO_2_ values estimated with each photodiode quartet are also calculated to assess the variability per source–detector distance. The CVs obtained with each subject are averaged and expressed as a mean±std for the three source–detector distances.

## 4. Results

Table 2 shows the estimated start of the far-field zone for the SFH 2703 green, red, and infrared LEDs using the formula for a rectangular source d=5.74·D. The sides of LEDs’ active areas and the rectangular package (Figure 2) are used as the *D* values for the calculations since the package can also influence the light emission, as detailed in Section LED Light Emission Characteristics.

### 4.1. Lab Experiment

**Figure 4:**
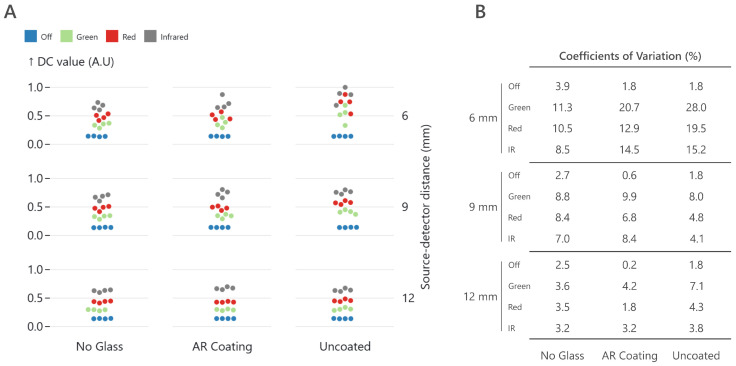
(**A**) Normalized DC values (0–1) recorded with the photodiodes rings (6 mm, 9 mm, and 12 mm) during the near- and far-field lab experiment. Results are sorted by source–detector distance (row) and optical window choice (column). Each row–column combination contains the readout of the four different photodiodes within a particular ring and glass choice using green, red, and infrared light and the LEDs off. (**B**) Coefficients of variation (CV) calculated for each quartet of photodiodes for a given wavelength, source–detector distance, and glass type.

Taking these estimated values as a reference, the measured DC values recorded with the three photodiode rings during the lab experiment are shown in Figure 4A. The results are presented using a dot plot, where each dot represents a DC amplitude. Dots are sorted in rows with increasing source–detector distance and in columns for the different optical windows. For each row–column cell, the values measured with each quartet of photodiodes are plotted in different colors for green, red, and infrared light, as well as for the off LEDs.

The first observation that can be made is that the DC values with the LEDs off are equal (dots forming a horizontal line) across all source–detector distances and glass types, which is reflected by the low CVs measured, below 5%. When looking at the 6 mm source–detector distance, the dots are no longer horizontal for all three wavelengths, indicating that the DC values differ depending on the selected photodiode. When looking at the CV (Figure 4B), this effect is more prominent for the uncoated glass, with a 28%, 19.5%, and 15.2% variability for green, red, and IR, respectively, than for the coated (20.7%, 12.9%, and 14.5%) and no glass (11.3%, 10%, and 8.5%).

The 9 mm ring already shows a minor variability, with dots being more horizontal. The minimum variability measured was 4.1% for infrared light using the uncoated glass and the maximal of 9.9% using green and coated glass. The 12 mm source–detector distance shows great readout stability across photodiode quartets, achieving quasi-horizontal lines for all wavelengths and glasses and CV similar to that measured with the LEDs off (<5%), except for green light using the uncoated glass. These lab results indicate that near- and far-field effects are present in PPG systems, with more variability observed when using green light.

### 4.2. PPG Signal Acquisition

**Figure 5:**
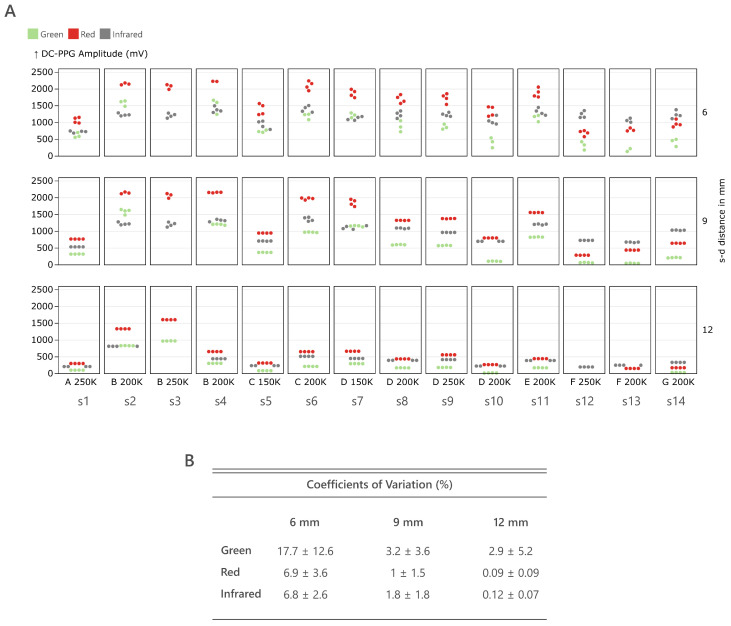
(**A**) Mean DC-PPG amplitudes recorded for all participants at 6 mm, 9mm, and 12 mm s–d distances using green, red, and IR. Subjects are sorted by skin type A–G, and the TIA feedback resistance is displayed next to it. (**B**) The mean and standard deviation of coefficients of variation are calculated for all subjects for each quartet of photodiodes for a given wavelength and source–detector distance.

The DC-PPG amplitudes obtained during the in vivo study are shown again using a dot plot, with amplitudes divided per subject, wavelength, and source–detector distance (Figure 5A). At 6 mm, the DC amplitudes of the same wavelength tend to vary, especially for green light, with amplitude variabilities observed in many subjects of up to 500 mV. This tendency is greatly reduced at 9 mm and fully gone at 12 mm, where the DC amplitudes of the same wavelength align horizontally. Wavelength-dependent amplitude differences can also be observed, especially at 6 and 9 mm. Red light amplitude predominates over green and infrared for subjects s1 to s11. For subjects s12 to s14, infrared shows a larger amplitude. This behavior is still observed at 12 mm but with closer amplitude values.

An effect of the skin type can be observed in the amplitude values of darker skin types (F and G) compared to lighter ones (B–E). For types B–E, signals oscillate on average between 1 and 2.5 V, while F and G oscillate between 0.1 and 1.5 V. At 12 mm, this tendency is still observed but greatly reduced, with amplitude values grouping together for the three wavelengths. A higher melanin content reduces the DC amplitudes registered with green, red, and infrared at 6, 9, and 12 mm of source–detector distance.

Overall, at 6 mm, dots are arranged more vertically for a given wavelength, while at 9 and 12 mm, they are more horizontal, showing more homogeneous amplitude values, which is supported by the coefficients of variation (Figure 5B), which decrease for all wavelengths with increasing source–detector distance. This behavior resembles the one observed in the lab setting, where green light shows again larger variability than red and infrared. These results indicate that near-field effects are present in PPG signals and generate random DC signal amplitudes when the source–detector distance is within the near-field.

**Figure 6:**
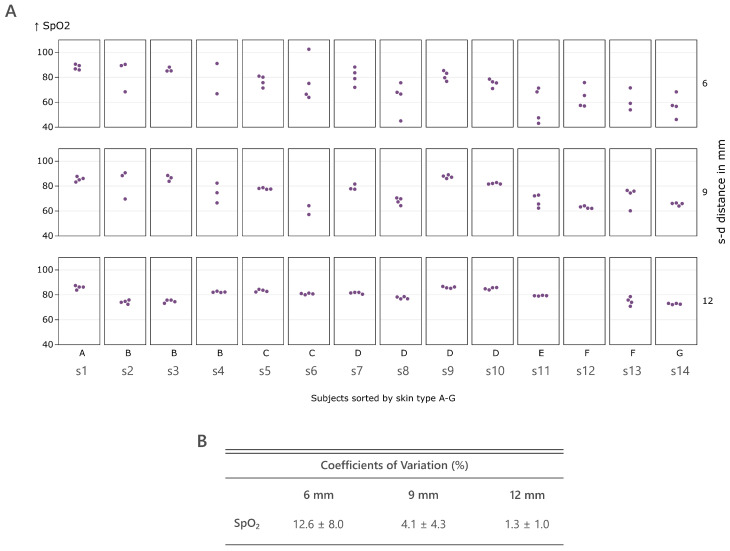
(**A**) SpO_2_ calculated from all photodiodes with varying source–detector distances (6 mm, 9 mm, and 12 mm) and spatial distribution. Participants are sorted by skin type A–G. (**B**) The mean and standard deviation of coefficients of variation of obtained SpO_2_ calculated for all subjects for each quartet of photodiodes for a given wavelength and source–detector distance.

Following the DC-PPG analysis, Figure 6A shows the SpO_2_ values estimated with each photodiode. While at 6 mm, great SpO_2_ disparities are observed for most subjects, at 12 mm, the SpO_2_ calculated with every optical pair is homogeneous for every subject. The DC-PPG amplitude differences described earlier at short source–detector distances impact the estimated SpO_2_, showing variabilities that are not caused by changes in oxygen concentration but by near-field-related effects. At 9 mm, the estimated values are stable for many subjects but still show significant disparities for others (s2, s4, s6, and s11). This behavior is further observed in the averaged CVs for the SpO_2_, which decrease with increasing source–detector distance (Figure 6B).

The results are significant and give a clear understanding of the impact of the DC amplitude variability at 6 mm caused by near-field effects on the SpO_2_ estimation.

## 5. Discussion

### 5.1. Lab Experiment

The lab experiment was conducted on a fixed optical table in a dark room to eliminate motion or ambient light influence. This setup left the instrumentation as the only additional noise source besides possible near-field effects. The results obtained with the LEDs off showed an equal readout for all photodiodes in all source–detector distances and glass combinations. This validates the study and the PPG system, as it guarantees that the observed disparities come from near- and far-field effects, not instrumentation-induced offsets. This first stage is necessary to correctly understand what is observed during the ‘real’ PPG acquisition.

When looking at the source–detector distances, clear variabilities were observed among the photodiodes at 6 mm from the source. These differences were found already in the no-glass readout for the three wavelengths. The use of glasses further increased the variability, being the highest for green light when using the uncoated glass (up to 28% of CV). These results suggest two things; first, a source–detector distance of 6 mm shows a high enough variability across photodiodes for all wavelengths to suggest that near-field effects are present at a source–detector distance larger than ≃ 3 mm, the largest start of the far-field region calculated using the LEDs’ active area only, as shown in Table 2. If the package area is taken for the calculation instead, at 6 mm, we are still inside the near-field region. This agrees with what was reported by Moreno et al. in [19], who state that the LED packaging influences and deforms light emission, making LEDs a non-Lambertian source and creating a near-field region that stabilizes into a far-field region at 9.2 mm from the source for the SFH 7013. The results obtained for the source–detector distances of 9 mm and 12 mm further validate this calculation, as the variability was reduced at 9 mm compared to the 6 mm but still higher than at 12 mm, where CVs were very similar to the ones measured with the LEDs off. The variability obtained at 9 mm could be explained as being in the boundary between the near- and far-field domains, where the radiance emission has not fully stabilized yet. All this indicates that the square side of the LED optical package should be taken as the *D* in the far-field calculation using the formula d=5.74·D.

A second observation is that the optical window worsens the near-field effects, especially the uncoated one. At 6 mm, the largest CVs change is observed for the green light, which goes from 11.3% (no glass) to 28% (uncoated), almost a three-fold variation. This tendency is also observed in red and infrared light but with a lower variability. For the 9 mm and 12 mm, which can be considered far-field, the glasses do not seem to have an impact, as the variabilities measured are similar to the no-glass ones, being even lower for some values. The larger variability observed with green light could be linked to the active area difference. While red and infrared have an active area of 0.3 × 0.3 mm, green is almost double, 0.5 × 0.5 mm. A larger active area could worsen the effect caused by the LED packaging, as light is more diffused and less concentrated.

### 5.2. PPG Signal Acquisition

The study with healthy participants showed that DC variabilities induced by near-field effects also affect PPG signals. The near-field behavior of LEDs can impact how light is absorbed and scattered in the skin at short source–detector distances. Since light is not yet homogeneously distributed, in this region, photodiodes placed at the same distance but at different spatial locations detect different amounts of light, giving different DC amplitude values. At larger distances, once in the far field, light has had the time to homogenize within the tissue, and the photodiodes detect similar DC values. These results complement and support those found in the lab experiment. Near-field effects are present in PPG signals and generate random DC signal amplitudes.

If the DC value is measured within this near field, unreliable estimation of physiological parameters dependent on the DC-PPG can be expected, as the random DC offset disparities caused by near-field effects cannot be compensated. This was demonstrated with the estimation of SpO_2_ with all photodiodes. An averaged SpO_2_ variability of 12.6% was measured using the 6 mm photodiodes, obtaining SpO_2_ values from 40% up to 100%. This was drastically reduced with the 12 mm photodiodes, where the CV was 1.3% with SpO_2_ values around 80%. These values do not represent the real oxygen saturation due to the use of the standard empirical equation instead of the calibration curve derived from a desaturation study but show the impact on the calculation of the near-field effect at 6 mm, while a great SpO_2_ estimation consistency is achieved at 12 mm.

These results consider a source–detector distance of 6 mm to be near-field. This distance and smaller ones are typically used in PPG systems in reflection mode, meaning that near-field effects can contribute to the PPG-DC component. This impact can be unnoticed since most systems only have one source and one detector. Near-field effects are probably negligible for pulse oximeters in transmission mode, as the source–detector distance is large enough to be in the far-field. On the contrary, wearables using PPG in reflection mode can be impacted by near-field effects, which would cause random DC-PPG offset disparities, leading to unreliable estimation of physiological parameters dependent on the DC-PPG, such as SpO_2_. These effects should be accounted for as part of the system calibration/characterization, especially when photodiode rings are used to derive physiological parameters, as is often the case in smartwatches. Understanding this is crucial for correctly designing robust PPG systems, capable of correctly estimating SpO_2_ by accounting for real oxygen saturation variabilities.

Several articles discuss the impact of the source–detector distance on the PPG signal, especially for multi-wavelength PPG [8,30]. These tend to focus primarily on the impact of the source–detector distance on the mean optical path and penetration depth of photons. All these studies take LEDs as an ideal light source, which cannot be considered as such given the short source–detector distances used and reported, which fall within the near-field regime. Simulations of photon migration in the skin should be conducted considering these near- and far-field regions.

Future work should validate these results using more variability of LEDs and source–detector distances to enlarge the characterization of near- and far-field effects on PPG systems. This study has used LEDs with a wide half-angle (65°). LEDs with smaller half-angles should also be included in future evaluations, as these small half-angles are achieved using a domed microlens to concentrate the light beam, which could alter the light emission and generation differently than the planar LED surface used by the SFH 7013 [15].

## 6. Conclusions

This work has shown that near-field effects are present in PPG systems configured in reflection mode. A distance of 9 mm has been calculated theoretically and experimentally as the start of the far-field region for the SFH 7013 multichip LED, commonly used in PPG applications. This study has characterized a common and well-known effect in optics, which, to the author’s knowledge, was not reported before for PPG applications.

PPG signals acquired in reflection mode with a source–detector distance smaller than 9 mm are subject to random DC variabilities caused by near-field effects. If these effects are not properly addressed, physiological parameters that rely on the DC-PPG component, such as SpO_2_, may be inaccurately estimated.

A methodology to identify the presence and influence of the near field in PPG signals has been proposed, which includes using the coefficient of variation as the signal quality index for near-field effects. This work contributes to a better understanding of PPG influencing factors associated with instrumentation choices. Despite the widespread use of the technology, multiple factors still compromise the signal quality and reliability. An individual understanding of influencing factors present in the selected application is crucial to advancing PPG technology and its robust and reliable use in existing and new applications.

## Figures and Tables

**Table 1:** Baseline demographic data of in vivo investigation involving 14 participants.

Demographic Data	Values
Age (y)	32.9±5.5
Gender (M/F)	13/1
BMI	25.1±3.5
Skin Tone (MST)	A(1), B(3), C(2), D(4), E(1), F(2), G(1)
Temperature (°C)	36.4±0.1
PR	69±11
SpO_2_	98.3±1.2

**Table 2:** Calculation of far-field start *(d)* for SFH 7013 LED following d=5.74·D using the active area side of each LED or the total LED package rectangle side as *D*.

	Active Area	LED Package
**Green**	2.9 mm	9.2 mm
**Red**	1.7 mm	9.2 mm
**Infrared**	1.7 mm	9.2 mm

## Data Availability

Data are contained within the article.
